# Fleece variation in alpaca (*Vicugna pacos*): a two-locus model for the Suri/Huacaya phenotype

**DOI:** 10.1186/1471-2156-11-70

**Published:** 2010-07-20

**Authors:** Silvano Presciuttini, Alessandro Valbonesi, Nolberto Apaza, Marco Antonini, Teodosio Huanca, Carlo Renieri

**Affiliations:** 1 Department of Physiological Sciences, University of Pisa, Via San Zeno 31, 56123 Pisa, Italy; 2 Department of Environmental Sciences, University of Camerino, Camerino, Italy; 3 INIA, ILLPA Puno, Rinconada Salcedo, Puno, Peru; 4 Ente per le Nuove Tecnologie, l'Energia e l'Ambiente, Roma, Italy

## Abstract

**Background:**

Genetic improvement of fibre-producing animal species has often induced transition from double coated to single coated fleece, accompanied by dramatic changes in skin follicles and hair composition, likely implying variation at multiple loci. Huacaya, the more common fleece phenotype in alpaca (*Vicugna pacos*), is characterized by a thick dense coat growing perpendicularly from the body, whereas the alternative rare and more prized single-coated Suri phenotype is distinguished by long silky fibre that grows parallel to the body and hangs in separate, distinctive pencil locks. A single-locus genetic model has been proposed for the Suri-Huacaya phenotype, where Huacaya is recessive.

**Results:**

Two reciprocal experimental test-crosses (Suri × Huacaya) were carried out, involving a total of 17 unrelated males and 149 unrelated females. An additional dataset of 587 offspring of Suri × Suri crosses was analyzed. Segregation ratios, population genotype frequencies, and/or recombination fraction under different genetic models were estimated by maximum likelihood. The single locus model for the Suri/Huacaya phenotype was rejected. In addition, we present two unexpected observations: 1) a large proportion (about 3/4) of the Suri animals are segregating (with at least one Huacaya offspring), even in breeding conditions where the Huacaya trait would have been almost eliminated; 2) a model with two different values of the segregation ratio fit the data significantly better than a model with a single parameter.

**Conclusions:**

The data support a genetic model in which two linked loci must simultaneously be homozygous for recessive alleles in order to produce the Huacaya phenotype. The estimated recombination rate between these loci was 0.099 (95% C.L. = 0.029-0.204). Our genetic analysis may be useful for other species whose breeding system produces mainly half-sib families.

## Background

After domestication, the primary breeds of fleece-producing mammals underwent a strong selection pressure to obtain a fiber with superior textile characteristics [1-3]. As a consequence, skin follicles and hair structures changed dramatically, following mutation accumulation [4]; for example, wild species with double coated fleece produced a variety of breeds with single coated fleece. Several fleece phenotypes are listed in Merino sheep (*Ovis aries*) [5], Angora and Cashmere goat (*Capra hircus*)[6], as well as in rabbit (*Oryctolagus cuniculus*)[6].

Two different types of fleece, Huacaya and Suri, are described in alpaca (*Vicugna pacos*), the domesticated variety of vicuña (*Vicugna vicugna*). Huacaya, by far the most common phenotype, is a single coated fleece characterized by compact, soft and highly crimped fibers, with blunt-tipped locks, which closely resemble those of Merino sheep. It is present in mummies from pre-Inca archaeological sites, suggesting that it was selected early from vicuña (which is double coated) for domestication. For this reason, it is often reported as the wild type, whereas Suri is thought to be derived from Huacaya through gene mutation, possibly with reduction of fitness [7]. It is a single coated fleece, less crimped, lustrous, with silky fibers. The locks are "cork-screw" shaped. It is very similar to mohair from Angora goat but it is not as bright. The Suri phenotype is easily detected at birth, and there is no evidence that it can be changed through a specific environment. Differences in cuticular scale frequency, follicular density, and secondary-to-primary follicular ratio have been reported [8-10].

The inheritance mode of the Suri/Huacaya phenotype is unclear. A hypothesis of Suri recessivity [7] has been abandoned in favor of a more widely accepted simple Suri dominance. Velasco [11] obtained 129 Huacaya offspring in Huacaya × Huacaya crosses, 9 Suri and 3 Huacaya in Suri × Huacaya crosses and 422 Suri and 89 Huacaya in Suri × Suri crosses. He proposed a single locus model with a dominant allele for Suri. Similar results were obtained by Ponzoni et al. [12]; Huacaya × Huacaya crosses resulted in 145 Huacaya and no Suri offspring, Suri sire × Huacaya dam resulted in 13 Huacaya and 11 Suri, and Suri × Suri resulted in 6 Huacaya and 29 Suri. They suggested a model with a single gene with two alleles, AIF_S _dominant over AIF_H_, though they also noted that the data were compatible with a model in which the trait was controlled by a group of closely linked alleles (haplotype). Baychelier [13] tested three inheritance hypotheses using the records of the Australian Alpaca Association Herd Book: one locus-two alleles, one locus-three alleles, and two loci (epistasis). According to the data the model of a single gene with three alleles can be rejected, whereas the two-gene model is more suitable than the one-locus model. Finally, Renieri et al. [14] tested four inheritance models in 588 offspring of 19 Suri × Suri segregating families, and the best data fitting model was one-locus-two alleles (Suri dominant). It should be noted that a small number of Suri offspring have been observed among the offspring of a large number of Huacaya × Huacaya crosses; for example, 12 Suri were born among 8,446 total offspring [15], and 3 Suri were born among 2,126 offspring [14]. In both studies, the Suri birth rate was 1.4 per thousand offspring.

The aim of the present work was to test the model of inheritance of Suri and Huacaya in controlled experimental crosses Suri × Huacaya. These are formally test-crosses, provided that one of the two phenotypes is fully recessive. As the alpaca is strictly monoparous, several females were mated with each male, giving rise to groups of half-sib families.

## Methods

### Animals

Two reciprocal experimental test-crosses were carried out: Suri males × Huacaya females and Huacaya males × Suri females. The trial involved a total of 17 unrelated males and 149 unrelated females (64 total Suri animals and 102 total Huacaya animals). Each F1 animal was born from a different female. The crosses were carried out at the experimental station of the INIA (the Peruvian National Institute for Agronomic Innovation) located in Quimsachata, Peru. The Station, comprising 6,282 hectares and situated at approximately 4400 m above sea level in the Santa Lucia District of the Puno Department, is committed to alpaca and llama breeding, conservation and genetic improvement. The alpaca herd of the Station was established about 10 years ago by collecting animals from dozens of different farms participating in the program "Conservation of Biodiversity of Domestic Camelids" of the Peruvian Ministry of Agriculture; since then, females were mated always to different, unrelated males to minimize inbreeding. The animals used in the present study were chosen from the pedigree registry of the Station as being unrelated to each other going back to the founding stock; this ensured that they were effectively unrelated.

A potential source of artifacts in segregation analysis of farm animals is false paternity, as a single (potentially false) offspring can determine the segregation status of the sire. In fact, this risk is negligible in planned crosses in alpaca, as the ovulation in this species is induced by mating and deposition of semen in the uterus. Without mating it is extremely rare to have a female ovulate, and females usually conceive after just one breeding [16]. Furthermore, males and females are kept separate from each other throughout the year and are brought together only for planned mating. The crosses were carried out by introducing a male to a female and controlling the mating process, which usually lasts a considerably long time.

We also analyzed a database of 587 offspring of 57 Suri males mated to Suri females [14]. This flock, consisting of Suri animals only, is raised at the Hacienda Mallkini (Michell Group), the largest private alpaca husbandry farm in Peru.

### Statistical analysis

#### Ascertainment correction

Two different methods to correct for ascertainment bias were used in this work, depending on the set of crosses being analyzed. A "complete selection model" was applied to the crosses where any number of Suri or Huacaya offspring could be observed, whereas a "truncate selection model" was applied to the crosses where only segregating male families were considered. Specifically, the probability *Pr*_*i*_*(r,s,p) *of observing *r *Huacaya offspring (*r *≥ 0 for complete selection, *r *≥ 1 for truncate selection) among *s *total offspring of the Suri male *i *mated to *s *Huacaya or Suri females, given the probability *p *of a Huacaya birth (constant among matings), was calculated either using the simple binomial probability distribution (complete selection) or the classical equation for truncate selection with random sampling of segregating families [17].

With both the complete and truncate selection models the total log-likelihood (*L*) of a set of matings *C *was then calculated as

$$L\left(C\right)=\sum_{i}ln\left[Pr_{i}\left(s,r,p\right)\right]$$

Here the probability *p *is a segregation ratio, for which the symbol *R *is used (*p *≡ *R*). The maximum likelihood estimate (MLE) of *R *was obtained by maximizing *L(C) *as a function of *R *using the Solver routine of Excel. The 95% confidence limits of the MLE of *R *were obtained by finding (again using Solver) the two values of R where the log-likelihood function was = *L*_*max *_- 5.0238/2, where the constant 5.0238 is the chi-square value with associated probability = 0.025 (this follows from the likelihood ratio test theory). If the likelihood function has a single maximum, there are two such values, on the left and on the right of the MLE of *R*, respectively

#### Heterogeneity of R values

For each male *i*, the two log-likelihood values *L1*_*i*_*(R1) *and *L2*_*i*_*(R2) *were calculated using the binomial function (simple or truncated as appropriate) by first setting two trial values for *R1 *and *R2 *(these are the two values of the segregation ratio that replace the single value *R*); then, the total log-likelihood (*L*) of a set of matings *C *was calculated as

$$L(C)=\sum_{i}max[L1_{i}(R1),L2_{i}(R2)];$$

the MLE of *R1 *and *R2 *were obtained by maximizing *L(C)*, using Solver. The value of *R1 *or *R2 *associated with the higher of the two values *L1*_*i *_*and L2*_*i *_was assigned to each male.

#### Monte Carlo analysis

To test for heterogeneity of the segregation ratio among Suri × Suri crosses, the observed segregation ratio in the total sample was used as the parameter value to calculate the binomial probability of observing *r *Huacaya offspring among *s *total offspring in each cross, and computed the total log-likelihood over all crosses. Ten thousand random permutations of the Huacaya offspring distributed among the observed sibships were then generated using the Poptools add-in of Excel http://www.cse.csiro.au/poptools/; the log-likelihood of each permutation was calculated, again using the binomial function. The proportion of times that the log-likelihood was lower than the value calculated for the true distribution was a direct estimate of the p-value of the null hypothesis that the number of Huacaya offspring was distributed according to a simple binomial distribution.

## Results

### Rejection of the single locus recessive model

In a first set of crosses, nine Suri males mated to Huacaya females produced a total of 94 offspring, and in the reciprocal crosses eight Huacaya males mated to Suri females produced 55 offspring. Table 1 shows the segregation of the Suri-Huacaya trait among the offspring of each male. Although both cross types are testcrosses (i.e., a dominant phenotype of unknown genotype is mated to a recessive phenotype), they are different, in that the Suri males can be mated repeatedly, whereas the Suri females can be mated only once per year. In the first case (Suri male), even a single Huacaya offspring shows that this individual is segregating, and this information can be used for all its offspring; in the second case (Suri female), in contrast, the segregating status of a female determined through a Huacaya offspring can be used only in that particular mating, as females produced one offspring only. For this reason, the segregation analysis must be conducted separately.

**Table 1:** Observed number of Suri and Huacaya offspring in reciprocal Suri × Huacaya crosses

A) SURI MALES			
Sire	Huacaya	Suri	Total
S0270100	3	6	9
S058104	2	9	11
S0810100	1	12	13
S237204	0	7	7
S244203	4	3	7
S443303	7	1	8
SEEI-024	6	9	15
SEEI-025	2	13	15
SSO 502	1	8	9
**Total**	**26**	**68**	**94**

**B) HUACAYA MALES**			
**Sire**	**Huacaya**	**Suri**	**Total**

S035104	0	6	6
S095101	3	3	6
S1199-M	4	5	9
S148102	3	6	9
S216204	0	3	3
S322203	3	6	9
S366203	1	7	8
S370397	2	3	5
**Total**	**16**	**39**	**55**
**Grand total**	**42**	**107**	**149**

Considering first the Suri males, only one out of nine animals did not segregate any Huacaya (among seven offspring). For the other eight animals it is possible to estimate the segregation ratio R of the Huacaya phenotype among their offspring assuming a truncated binomial distribution. With this model the maximum likelihood estimate of R was 0.290, with 95% C.L. = 0.184-0.409. Clearly, a value of R = 0.5, which is expected for a testcross of a recessive single-locus model can be rejected.

With regard to the reciprocal cross a similar analysis cannot be carried out. When one offspring only can be obtained from a Suri animal (the female in our case), the observed segregation ratio for a number of animals of unknown genotype is a function of two parameters: 1) the probability R that a segregating animal generates a Huacaya offspring (e.g., the Mendelian transmission probability), and, 2) The probability H that an animal is segregating. Most value combinations of these two parameters produce the same value of the maximum likelihood. Therefore, a practical approach could be to estimate the value that each parameter assumes, once the other parameter is fixed on a value representing a reasonable choice. For example, if we assume that the segregation ratio R is 0.5 (the probability that a Suri female mated to a Huacaya male produces a Huacaya offspring for a simple recessive genetic model), the maximum likelihood estimate of H is 0.582. Conversely, if we assume that H among the Suri females has the same value as that observed among Suri males (= 8/9, or 0.88), the maximum likelihood estimate of R is 0.331. The similarity of this last value with that estimated for the crosses of Suri males is certainly interesting.

Two unexpected conclusions can be drawn from these results. The first is the rejection of the single-locus recessive model for the Huacaya phenotype, the second is that a considerable proportion, approximately 58% in the most conservative scenario, of the Suri animals are estimated to be segregating. This is unexpected, as most breeders would consider their Suri alpaca herds as almost pure for this phenotype as a consequence of their careful breeding strategy.

### Heterogeneity of segregation ratio among males

The results of the cross Suri males × Huacaya females were examined more closely. The segregation ratio seems to be heterogeneous among different males, the extremes being the two males S0810100 and SSO-502 (with one Huacaya in 13 offspring and 1 Huacaya in 8 offspring, respectively), and, on the opposite side, the two males S443303 and S244203 (with seven Huacaya in 8 offspring and four Huacaya in 7 offpring, respectively). In fact, the G-square statistics of the contingency table was highly significant for heterogeneity (G-square = 28.1, 8 d.f, P < 0.001). This result justified an analysis in which two hierarchical models were contrasted: 1) the model in which a single parameter R was common to all males; 2) a model in which some males segregated Huacaya offspring at ratio R1 and the other males segregated at ratio R2. The maximum likelihood estimate of R in the first case has already been shown (R = 0.290, log likelihood = -20.77). For the second model (only the eight sires with at least one Huacaya offspring were included), the calculated maximum likelihood value separated the first four males, with an estimate of R1 = 0.08, from the other four males, with an estimate of R2 = 0.51, and total log likelihood = -11.76. Since minus twice the log likelihood difference between a model and a more general model is approximately distributed as χ^2^, with degrees of freedom determined by the difference in the number of estimated parameters (one in the present case), the first model was rejected in favor of the second (P < 0.001).

### A model of two linked loci

A hypothesis that could explain these unexpected results is that the Huacaya phenotype is determined by the joint homozygosity for recessive alleles at two linked loci, and the Suri phenotype is determined by the presence of a dominant allele at either locus. In this model the Huacaya-segregating crosses of the present work would be of the type AB/ab × ab/ab (or even Ab/ab × ab/ab, or aB/ab × ab/ab; for the sake of simplicity, we refer here only to the double heterozygous individuals). We use a notation in which a single slash means a genotype with phase unknown, and a double slash means a phase-known genotype, or a diplotype. The double heterozygous animals can be either AB//ab or Ab//aB; if the two loci are tightly linked, only the first diplotype can segregate Huacaya offspring in a test-cross (at a ratio 1:1), and the model is indistinguishable from a single-locus recessive model. If, on the other hand, the two loci are separated by a recombination fraction *h*, both diplotypes can originate Huacaya offspring, the AB//ab diplotype at the ratio R1 = (1/2 - *h*) Huacaya: (1/2 + *h*) Suri, and the Ab//aB diplotype at the ratio R2 = 1/2*h *Huacaya: (1 - 1/2*h*) Suri. In fact, if the real Suri males of our crosses are an admixture of the two double heterozygous diplotypes, and if *h *is not too small, we expect to observe precisely the results of Tab 2, with a fraction of the males producing Huacaya offspring at a ratio close to 1:1 and the other fraction producing Huacaya offspring at a ratio close to 0:1.

The above hypothesis prompted us to estimate the recombination fraction *h *from the data. Assuming that the first four males of Tab. 2 segregated Huacaya offspring at ratio 1/2 *h *and Suri offspring at ratio 1 - 1/2 *h*, and the other four males segregated at ratios 1/2 - *h *and 1/2 + *h*, respectively, the maximum likelihood estimate of *h *resulted = 0.099, with 95% C.L. = 0.029-0.204.

### Analysis of Suri × Suri crosses

In order to further test this double recessive model of two linked loci using additional independent data, we reconsidered the results of our previous analysis based on 587 offspring of 57 Suri males mated to Suri females [14]. The segregation of the Huacaya trait in this series is shown in Table 2. Among the 57 males, 34 did not produce any Huacaya offspring, but most of them were not very informative due to their small progeny size (first data row in Table 2). Setting up a minimum of 10 offspring per animal, we find 7 total non-segregating and 15 total segregating animals (68% of segregating animals). If the minimum is placed at 20 offspring per animal, there are 3 non-segregating and 9 segregating animals (75% segregating), and for a minimum of 30 offspring per animal there is one non-segregating and 4 segregating animals (80% segregating animals). The mean value, weighted by the number of males, was 73.8%. In conclusion, a remarkably high proportion of segregating animals among Suri males is confirmed by these data.

**Table 2:** Segregation of the Huacaya phenotype among half-sib families from Suri × Suri crosses

	Number of offspring per male																					
#Huacaya	1	2	3	4	5	6	7	8	9	10	12	18	19	20	22	24	27	31	32	34	45	Total
0	21	1	2		1		1	1		2		1	1		1	1				1		34
1	1				1	1		1		1				1			3		1			10
2				1	2				1	1	1	1										7
3													1					1				2
4																	1					1
6													1							1		2
11																					1	1

Total males	22	1	2	1	4	1	1	2	1	4	1	2	3	1	1	1	4	1	1	2	1	57

Total Huacaya	1	0	0	2	5	1	0	1	2	3	2	2	9	1	0	0	7	3	1	6	11	57
Total offspring	22	2	6	4	20	6	7	16	9	40	12	36	57	20	22	24	108	31	32	68	45	587

The "#Huacaya" column lists all instances in which that number of Huacaya offspring was observed in sibships whose sizes are listed in the row under "Number of offspring per male".

**Figure 1:**
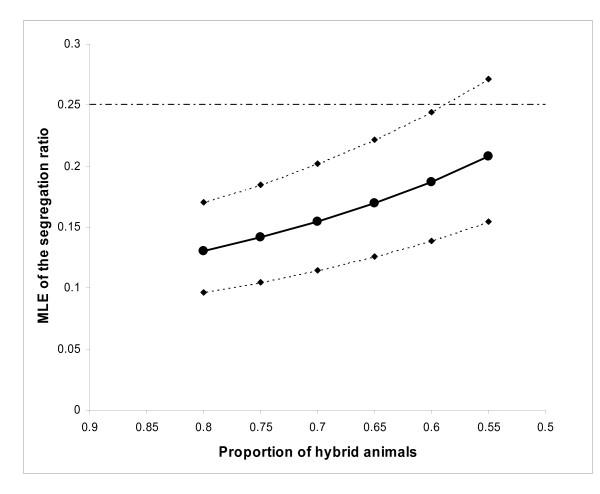
**Expected fraction of Huacaya offspring**. Huacaya segregation ratio in Suri × Suri crosses. Thick line: MLE of the segregation ratio R as a function of the proportion of segregating animals in the population. Dotted lines: 95% confidence limits of R.

We estimated the segregation ratio of the Huacaya phenotype in this series by maximum likelihood. Considering the entire dataset, the probability that any cross generated a Huacaya offspring depended on the chance that both mates were segregating and on the segregation ratio R. For the females the segregating condition (H) was always unknown, whereas the segregating males were identified by their (first) Huacaya offspring. Therefore, two values of the parameter *p *of the binomial distribution for the number r of Huacaya offspring among sibships of size *s *were used: 1) p_1 _= H·H·R for the non-segregating crosses and, 2) p_2 _= H·R for the segregating crosses. Figure 1 shows the MLE of the segregation ratio R for increasing values of the proportion of segregating animals among the population of mates. It may be seen that a Mendelian segregation ratio of a recessive trait (0.25) is included within the 95% confidence limits of the MLE of R only when the proportion of segregating animals is lower than ca. 0.57; for a proportion of segregating animals compatible with the value estimated from data (0.738), the single-locus recessive model is strongly rejected. Our previous analysis, in which the single-locus recessive model was not rejected, was based on the implicit assumption of a 50:50 proportion of AA: Aa genotypes among the females mated to obligate heterozygous males (selected with at least a Huacaya offspring). Repeating this analysis using a MLE approach (truncated ascertainment model), the following results were obtained: for H = 0.5, R = 0.224 (95% CL 0.172 - 0.330); for H = 0.74, R = 0.165 (95% CL 0.116 - 0.223). Thus, once again, the single-locus recessive model proves to be compatible with the data only for values of H that are incompatible with the observed proportion of segregating males.

Furthermore, possible evidence of heterogeneity of the segregation ratio R among the segregating males was examined. For example, an animal produced 11 Huacaya among 45 offspring and another male produced a single Huacaya among 32 offspring. To test the null hypothesis of homogeneity of R among males we applied a permutation test, generating random samples of the 57 Huacaya offspring distributed among the 23 segregating males. Among 10,000 Monte Carlo replicates, 9,914 produced a likelihood value larger than the value calculated from the real data (P = 0.0086), thus rejecting the null hypothesis of homogeneity of the segregation ratio. Again, this result justified contrasting the MLE of two models, the first based on a single parameter (R), the second based on two parameters (R1 and R2). In the first case, the MLE of R was 0.140, with log-likelihood = -42.4. In the second case, the values of R1 and R2 were 0.216 and 0.057, respectively, with log-likelihood = -31.2. Therefore, the first model is strongly rejected. The data are compatible with a model in which a proportion of Suri × Suri crosses segregate Huacaya offspring at a ratio slightly lower than 0.25 and the rest of the crosses segregate at a ratio slightly above zero. This supports the theory of homozygosity at two linked loci as the basis of the Huacaya phenotype.

## Discussion

The model of a single-locus recessive allele for the Huacaya phenotype has been supported by several studies in the past. We also addressed this problem in a previous analysis [14] using a large dataset from a single farm that keeps detailed record of the offspring from Suri × Suri crosses. In this analysis the single-locus recessive model was not rejected. However, the authors were not satisfied with the results, as the situation was not completely convincing. A set of experimental testcrosses (Suri × Huacaya) were thus set up in a controlled environment, of which the results are presented here.

The most important conclusion of this work is that the single-locus, fully recessive model can be rejected. The ratio Suri-Huacaya in the offspring is significantly higher than the expectation 1:1. Looking for a possible explanation, we made two unexpected observations: 1) the proportion of segregating animals, i.e. those that had produced at least one Huacaya offspring, was remarkably large, even in breeding conditions where one would expect the Huacaya trait to be almost eliminated; 2) the segregation ratio, despite being on the whole lower than expected, appeared to be heterogeneous among the segregating animals. Most of the analyses of the present paper were devoted to supporting these two observations statistically.

Considering the proportion of segregating animals, the nine Suri males mated to Huacaya females were randomly chosen from a herd of dozens of animals, thus roughly representing the general situation of a farm where genetic improvement is actively practiced. Nonetheless, eight out of nine males are segregating, and even the last animal has still a chance of being segregating, as only seven offspring were obtained from it at the moment of this survey. For the Suri females mated to Huacaya males, the estimate of the proportion of segregating animals is more difficult, as only one offspring can be observed for each female. However, the fact that among 55 females 16 (29%) produced a Huacaya offspring shows that the proportion of segregating animals is very large (>50%). We addressed the same question in the database of our previous work, and reached the conclusion that ca. 75% of the animals of a large (>2000 animals), carefully bred herd, are segregating for the Huacaya trait. Such a high proportion of segregating animals is hardly compatible with the segregation of a single locus. For example, at generation 0 ("P" in Mendelian terms) a breeding farm selects a certain number of Suri animals from several sources to establish a genetically improved herd; to be extremely conservative we may assume that all these individuals are heterozygous for the Suri dominant allele (i.e., they are all Ss). Therefore, the F1 offspring genotype distribution is 25% SS, 50% Ss, and 25% ss (Huacaya). The Huacaya F1 animals are discarded from reproduction so that the proportion of segregating Ss animals among the F1 reproducing animals is 2/3. This is essentially the selection scheme applied to the Suri flock raised at the Mallkini farm for more than 10 years: all Huacaya offspring born in the herd are removed. If this process is repeated over the generations, the proportion of heterozygous individuals among phenotypically Suri animals declines according the series 2/3, 1/2, 2/5, 1/3, 2/7, 1/4, 2/9, ... Thus, after only four generations, the proportion of heterozygous animals (1/3) is expected to be substantially lower than the proportion of homozygous Suri animals. Despite the simplified assumptions, the observations are clearly conflicting with expectations.

A process that may distort the segregation ratios from the Mendelian expectations is selection. In fact, a reduction of fitness has been suggested for the "mutant" Suri animals in comparison with the Huacaya wild type. For example, the apparent excess of segregating animals may be due to an unperceived bias, like *in utero *selection against the homozygotes for a dominant Suri allele. This hypothesis was falsified by the Suri × Suri crosses, as it predicts a proportion of Huacaya offspring higher than 25%, whereas the opposite was true. We explored several other possibilities implying unintentional selection of different genotypes, but none was compatible with the data.

The second observation, the heterogeneity of the segregation ratio among animals, was clearly suggested by the results of the Suri males × Huacaya females (contingency table). Consequently, a maximum likelihood approach showed that a model with two different ratios was significantly better than a model with a single parameter, both in the dataset of the present work and in the larger database of our previous work. In both cases, one of the values was similar to or slightly lower than the expected segregation ratio for a Mendelian single locus cross (1:1 in the first case, 1:3 in the second case), whereas the second value was slightly above zero.

A parsimonious genetic model explaining these facts implies two linked loci that must be both homozygous for a recessive allele in order to produce the Huacaya phenotype. If these loci are tightly linked, the situation cannot be distinguished from a single-locus recessive model, and this can justify why this model has not been rejected in the previous analyses. However, if the loci are moderately linked, the double heterozygous (Suri) animals will produce recessive gametes ("Huacaya haplotypes") at two different ratios, depending on the cis/trans configuration of their diplotype. When both recessive alleles are in cis, a Huacaya haplotype will be transmitted with 50% chance minus the recombination rate (which restores a Suri haplotype); when the recessive alleles are in trans, only recombination may produce Huacaya haplotypes, so that their transmission has a probability much lower than 50%. The estimated recombination fraction was about 10%, although this estimate is rather speculative given the low number of informative meioses, so that the confidence interval was rather large (3% to 20%).

## Conclusions

In conclusion, both the data of the experimental test-crosses conducted in the present work and the data of a large independent set of Suri × Suri crosses show that the previously suggested single locus recessive model for the Suri/Huacaya phenotype is unacceptable. In contrast, the data are compatible with the hypothesis that the Huacaya phenotype derives from homozygosis of recessive alleles at two linked loci. We suggest that this is the most parsimonious model that can explain the results. This genetic model, if proven true, may be useful to dissect the genetics of fleece phenotypes in other species of commercial significance. In addition, the genetic analysis may be useful for animals whose breeding systems produce large half-sib families.

## Appendix

**Figure 2:**
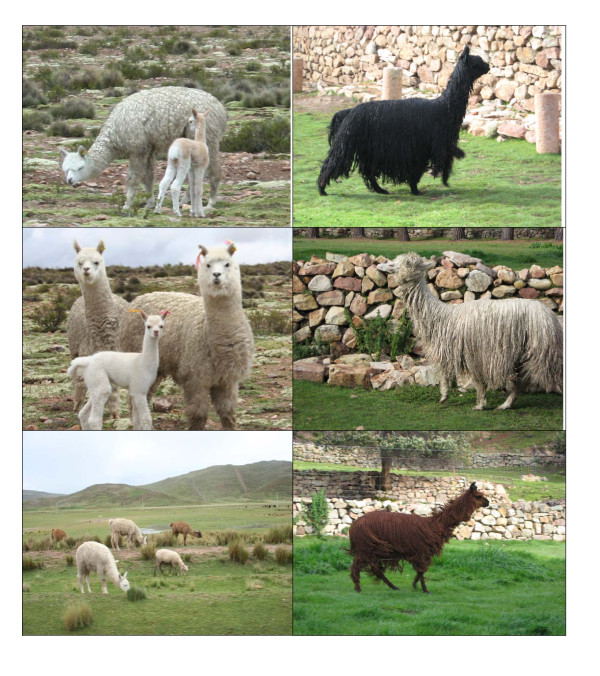
**Huacaya and Suri phenotypes**. The left column show some usual Huacaya alpacas; the right column show three magnificent Suri champions (Photo by S.P.).

Pictures in figure 2 show some alpaca with Huacaya and Suri phenotype

## Authors' contributions

SP conducted the statistical analysis and drafted the manuscript. AV participated in the design of the study and contributed to the statistical analysis. NA and TH organized the experimental crosses and registered the results. MA contributed to the design of the study. CR conceived of the study, participated in its design and coordination and helped to draft the manuscript. All authors read and approved the final manuscript.
